# Estimation of periodontal pocket surface area in small to medium dogs: a proof-of-concept study

**DOI:** 10.1186/s12917-021-03116-0

**Published:** 2022-01-03

**Authors:** Kazuya Tamura, Masako Tokuzen-Tai, Yasir Dilshad Siddiqui, Hitomi Tamura-Naito, Yoshiharu Nagahara, Kazu Hatanaka-Takeuchi, Tadashi Yamamoto, Shogo Takashiba

**Affiliations:** 1 Department of Pathophysiology-Periodontal Science, Okayama University, Graduate School of Medicine, Dentistry and Pharmaceutical Sciences, 2-5-1, Shikata-cho, Kita-ku, Okayama City, 700-8525 Japan; 2 Tamura Animal Clinic, Okayama City, Okayama, Japan; 3 Department of Periodontics and Endodontics, Okayama University Hospital, Okayama City, Okayama, Japan; 4 Department of Neural and Pain Sciences, School of Dentistry, University of Maryland, Baltimore, USA; 5 Nagahara Animal Hospital, Okayama City, Okayama, Japan

**Keywords:** Dog, Periodontitis, Periodontal pocket surface area, Estimation method, Periodontology

## Abstract

**Background:**

Periodontal disease is the most common dental disease in dogs. Although the systemic effects of periodontal disease have not been clarified in veterinary science, it is necessary to evaluate the effects of periodontal disease in clinical trials in the future. There have been a few clinical attempts made, however, to assess the severity of periodontal inflammation and its impact on the systemic health of dogs. Meanwhile, in the field of dentistry for humans, the periodontal inflamed surface area (PISA) and periodontal epithelial surface area (PESA) have been used to quantitatively assess the degree of periodontal disease affecting a single tooth as well as the overall extent of periodontitis. Recent studies have also suggested the use of these assessments to examine the relationship between periodontal inflammation and systemic health.

**Results:**

The estimation formula for a dog’s periodontal pocket surface area (PPSA), an alternative to PISA and PESA in humans, was established using body weight and periodontal pocket depth. Actual values were measured using extracted teeth from various dog breeds and sizes (2.3–25.0 kg of body weight) to obtain universal regression equations for PPSA. Altogether, 625 teeth from 73 dogs of 16 breeds were extracted and subsequently analyzed for morphological information.

PPSA was measured in 61 dogs of 10 breeds with periodontal disease using the established estimation formulas, and the correlation between PPSA and preoperative blood chemistry data was analyzed accordingly. A strong correlation was found between PPSA and serum globulin (*r* = 0.71) while moderate correlations were found for C-reactive protein (*r* = 0.54) and serum albumin (*r* = -0.51).

**Conclusions:**

Estimation formulas using body weight and the 6-point probing depth were established for determining PPSA. Direct correlations between PPSA and several blood test results were observed in the study sample. Taken together, these results suggest that PPSA could be useful for evaluating the effects of periodontitis on systemic conditions in dogs.

**Supplementary Information:**

The online version contains supplementary material available at 10.1186/s12917-021-03116-0.

## Background

One of the common problems in veterinary patients is periodontal disease [1]. Clinical data from a primary veterinary practice in England suggested a prevalence rate of periodontal disease of 9.3%, which ranked as the second highest in canine diseases [2]. Up to the age of 2 years, 80% of dogs have some form of periodontal disease [3]. The World Small Animal Veterinary Association Global Dental Guidelines [4] suggest that periodontal disease in dogs may influence systemic diseases such as diabetes, malignant tumors, early death, and chronic inflammation. The affected organs include the liver, kidney, heart, and lungs. In addition, several veterinary clinical studies have suggested systemic effects of periodontal disease [5–8]. Some of these studies have been based on results of human studies [9–12]. However, the assessment of the extent of periodontal disease in many clinical trials was not quantitative.

Recently, in the dentistry field for humans, the periodontal inflamed surface area (PISA) and periodontal epithelial surface area (PESA) [13] have been used to quantitatively assess the degree of extent of lesions and inflammatory infiltration of periodontal disease in the complete oral unit. PESA is the total surface area of the periodontal pocket epithelium in one oral unit, whereas PISA represents PESA with bleeding on pocket probing. Several studies have evaluated periodontal disease using PISA and PESA to examine its relationship with systemic diseases [14, 15]. The loss of periodontal attachment in dogs has also been evaluated [16]. Although the evaluation method adequately assessed the extent of periodontal disease, it was assessed with a unique score and was not expressed using an international system of units.

In this study, a method was established to estimate the periodontal pocket surface area (PPSA) after periodontal pocket probing as a clinical alternative to PESA in humans. In the process of establishment, a forecasting formula was considered corresponding to the variety of body sizes and breeds of dogs. Furthermore, this method was applied to dogs with periodontal disease in a small-scale clinical trial to evaluate whether the severity of periodontitis assessed using PPSA related to the blood chemistry data.

## Results

### Animal and sample collection

Altogether, 625 teeth were extracted from 73 dogs of 16 breeds. Their body weights ranged from 2.3 to 25.0 kg (median: 5.6 kg). The distribution of extracted tooth number, root length, and body weight of each breed is listed in Supplement 1 and summarized in Table 1.

**Table 1:** Distribution of number of extracted teeth and body weight of each breed

Breed	Number of teeth	Number of dogs	Range of weight (kg)
Miniature Dachshund	325	36	2.3–8.5
Toy Poodle	116	12	2.3–6.0
Papillon	41	4	5.2–7.0
Chihuahua	26	3	3.4–4.0
Yorkshire Terrier	26	2	3.0–6.1
Maltese	26	2	3.0–3.5
Shih Tzu	19	2	6.5–7.5
Pomeranian	12	1	3.5
Italian Greyhound	7	1	5.8
Jack Russell Terrier	7	1	4.8
Miniature Schnauzer	6	2	7.6–8.6
Beagle	6	1	8.3
Mongrel	3	3	5.3–13.8
French Bulldog	2	1	9.6
Siba	2	1	16.4
Labrador Retriever	1	1	25.0
Total	625	73	2.3–25.0

Summary table of Supplement 1

### Correlation between actual root length and body weight

**Fig. 1:**
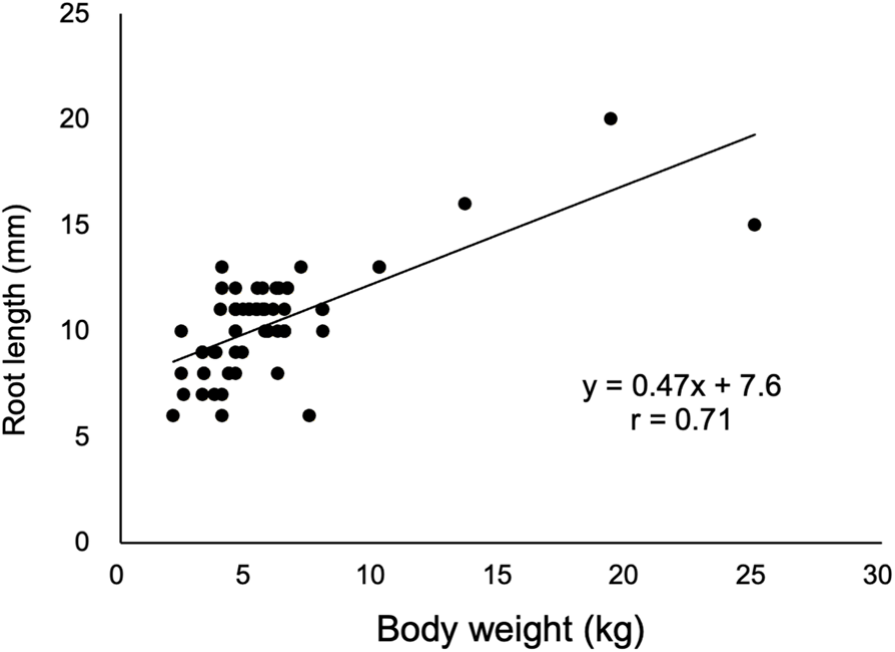
Scatter plot for root length of the maxillary fourth premolar and body weight Linear regression formula and correlation coefficient are shown (*n* = 62). The estimation formula is y = 0.47x + 7.6 (*r* = 0.71)

For each tooth type, the actual root length and body weight were compared (Supplement 2). A strong correlation (*r* ≥ 0.7) was observed with the maxillary fourth premolar (Max P4) at 0.71 (*p* < 0.01; Table 2). The scatter plot and regression equation for Max P4 are shown in Fig. 1.

**Table 2:** Correlation coefficient between actual root length and actual body weight for each tooth type

Maxillary tooth (*n* = 334)	Correlation coefficient	Determination coefficient	*p*-value	Mandibular tooth (*n* = 291)	Correlation coefficient	Determination coefficient	*p*-value
max I1 (*n* = 20)	0.27	0.07	0.26	mandi I1 (*n* = 26)	0.33	0.11	0.1
max I2 (*n* = 20)	0.30	0.09	0.20	mandi I2 (*n* = 35)	0.45	0.20	< 0.01
max I3 (*n* = 48)	0.30	0.09	< 0.05	mandi I3 (*n* = 42)	0.42	0.18	< 0.01
max Canine (*n* = 34)	0.51	0.26	< 0.01	mandi Canine (*n* = 24)	0.51	0.26	< 0.05
max P1 (*n* = 34)	0.36	0.13	< 0.05	mandi P1 (*n* = 10)	0.13	0.02	0.69
max P2 (*n* = 27)	0.57	0.32	< 0.01	mandi P2 (*n* = 30)	0.53	0.28	< 0.01
max P3 (*n* = 16)	0.68	0.46	< 0.01	mandi P3 (*n* = 33)	0.51	0.26	< 0.01
max P4 (*n* = 62)	0.71^a^	0.50	< 0.01	mandi P4 (*n* = 25)	0.46	0.21	< 0.05
max M1 (*n* = 46)	0.30	0.09	< 0.05	mandi M1 (*n* = 36)	0.38	0.14	< 0.05
max M2 (*n* = 27)	0.66	0.43	< 0.01	mandi M2 (*n* = 22)	0.64	0.40	< 0.01
				mandi M3 (*n* = 8)	0.42	0.18	0.30

Summary table of Supplement 2

*max* maxillary, *mandi* mandibular, *I* incisor, *P* premolar, *M* molar, *n* number of extracted teeth. ^a^Significant correlation (*r* ≥ 0.7)

Summary table of Supplement 2. Additionally, see Fig. 1 for the formula for the root length of max P4 (y = 0.47x + 7.6)

### Correlation between actual root length and estimated value using the formula for max p4 root length

For each tooth type, the actual root length and estimated value were compared using the formula for max P4 root length (Supplement 3). A strong correlation (*r* ≥ 0.7) was observed for the four types of teeth: maxillary second molar, mandibular canine, mandibular first premolar, and mandibular third molar (Table 3). Moderate correlation (0.7 > *r* ≥ 0.5) was observed for the maxillary second premolar, maxillary third premolar, mandibular first incisor, mandibular second incisor, mandibular second premolar, mandibular third premolar, and mandibular fourth premolar (Table 3). Furthermore, a weak correlation (0.5 > *r* ≥ 0.3) was observed for nine types of teeth (Table 3).

**Table 3:** Correlation coefficient and regression equation for actual root length of each tooth type and estimated value using the max P4 root length formula

Maxillary tooth	Correlation coefficient	Determination coefficient	Regression equation	*p*-value	Mandibular tooth	Correlation coefficient	Determination coefficient	Regression equation	*p*-value
max I1	0.46	0.21	z = 1.06y-3.63	0.22	mandi I1	0.58^b^	0.34	z = 0.64y + 2.55	0.06
max I2	0.34	0.12	z = 0.50y + 3.00	0.40	mandi I2	0.52^b^	0.27	z = 0.86y + 0.26	< 0.05
max I3	0.49	0.24	z = 0.94y + 1.60	< 0.05	mandi I3	0.37	0.14	z = 0.59y + 4.52	0.07
max Canine	0.49	0.24	z = 1.49y + 2.36	< 0.01	mandi Canine	0.71^a^	0.50	z = 1.13y + 5.92	< 0.01
max P1	0.34	0.12	z = 0.34y + 2.69	0.21	mandi P1	0.76^a^	0.58	z = 0.29y + 3.29	0.45
max P2	0.63^b^	0.40	z = 0.70y-0.96	< 0.05	mandi P2	0.52^b^	0.27	z = 0.41y + 2.83	< 0.05
max P3	0.53^b^	0.28	z = 0.37y + 2.52	0.06	mandi P3	0.60^b^	0.36	z = 0.45y + 3.15	< 0.05
max P4	ND	ND	ND		mandi P4	0.52^b^	0.27	z = 0.52y + 3.99	0.07
max M1	0.45	0.20	z = 0.44y + 2.95	< 0.01	mandi M1	0.47	0.22	z = 0.78y + 3.20	< 0.05
max M2	0.70^a^	0.49	z = 0.47y-1.44	< 0.01	mandi M2	0.33	0.11	z = 0.25y + 3.65	0.24
					mandi M3	0.82^a^	0.67	z = 0.82y-5.36	0.18

Summary table of Supplement 3

*max* maxillary, *Mandi* mandibular, *I* incisor, *P* premolar, *M* molar, *ND* not determined as treated as standard

^a^strong correlation (*r* ≥ 0.7); ^b^moderate correlation (0.7 > *r* ≥ 0.5)

z: estimated root length of each tooth, y: root length of each tooth estimated using the formula shown in Fig. 1

### Correlation between actual root surface area and estimated root length

For each tooth type, the actual root surface area and estimated root length were compared using the formula for max P4 root length (Supplement 4). A weak correlation (0.5 > *r* ≥ 0.4) was observed for two types of teeth, namely, maxillary second premolar and mandibular first premolar (Table 4). A moderate correlation (0.7 > *r* ≥ 0.5) was observed for three types of teeth: maxillary first incisor, mandibular second incisor, and mandibular fourth premolar (Table 4). Furthermore, a strong correlation (*r* ≥ 0.7) was observed for the remaining 16 types of teeth (Table 4).

**Table 4:** Correlation coefficient and regression equation obtained from a scatter plot of the root length and the root surface area

Maxillary tooth	Correlation coefficient	Determination coefficient	Regression equation	*p*-value	Mandibular tooth	Correlation coefficient	Determination coefficient	Regression equation	*p*-value
max I1	0.68^b^	0.46	α = 12.1z + 16.6	< 0.01	mandi I1	0.80^a^	0.64	α = 13.5z + 1.6	< 0.01
max I2	0.80^a^	0.64	α = 23.2z-45.7	< 0.01	mandi I2	0.54^b^	0.29	α = 14.8z + 2.0	< 0.01
max I3	0.81^a^	0.66	α = 31.9z-89.5	< 0.01	mandi I3	0.72^a^	0.51	α = 23.3z-77.7	< 0.01
max Canine	0.79^a^	0.62	α = 26.9z + 6.30	< 0.01	mandi Canine	0.90^a^	0.81	α = 40.6z-107.3	0.15
max P1	0.73^a^	0.53	α = 24.4z-32.6	< 0.01	mandi P1	0.49	0.24	α = 30.5z-24.6	< 0.01
max P2	0.46	0.21	α = 13.7z + 105.7	< 0.05	mandi P2	0.74^a^	0.55	α = 43.4z-87.9	< 0.01
max P3	0.83^a^	0.69	α = 70.5z-196.7	< 0.01	mandi P3	0.79^a^	0.62	α = 46.1z-117.6	< 0.01
max P4	0.80^a^	0.64	α = 119.2y-560.7	< 0.01	mandi P4	0.69^b^	0.48	α = 36.5z-45.7	< 0.01
max M1	0.72^a^	0.52	α = 89.8z-219.4	< 0.01	mandi M1	0.77^a^	0.59	α = 71.4z-113.3	< 0.01
max M2	0.76^a^	0.58	α = 39.4z-33.9	< 0.01	mandi M2	0.88^a^	0.77	α = 45.4z-118.0	< 0.01
					mandi M3	0.91^a^	0.83	α = 17.7z + 10.8	0.27

Summary table of Supplement 4

*max* maxillary, *Mandi* mandibular, *I* incisor, *P* premolar, *M* molar

^a^strong correlation (*r* ≥ 0.7); ^b^moderate correlation (0.7 > *r* ≥ 0.5)

y: estimated root length for max P4, z: estimated root length for others, α: estimated root surface area

### Establishment of ppsa estimation formula (Table 5)

**Table 5:** Regression equations obtained from Supplements 2, 3, 4, and 5

**A: Maxilla**				
	Estimated root length		Estimated root surface area	PPSA
	Supplement 2	Supplement 3	Supplement 4	Supplement 5
max I1		1.06 × Root length (max P4)-3.63	12.1 × Root length (max I1) + 16.6	Estimated surface area × {1- (Estimated root length—Average PD_1-6_)^2^ / Estimated root length ^2^}
max I2		0.50 × Root length (max P4) + 3.00	23.2 × Root length (max I2)-45.7	
max I3		0.94 × Root length (max P4) + 1.60	31.9 × Root length (max I3)-89.5	
max Canine		1.49 × Root length (max P4) + 2.36	26.9 × Root length (max Canine) + 6.30	
max P1		0.34 × Root length (max P4) + 2.69	24.4 × Root length (max P1)-32.6	
max P2		0.70 × Root length (max P4)-0.96	13.7 × Root length (max P2) + 105.7	
max P3		0.37 × Root length (max P4) + 2.52	70.5 × Root length (max P3)-196.7	
max P4	0.47 × BW + 7.6	ND	119.2 × Root length (max P4)-560.7	
max M1		0.44 × Root length (max P4) + 2.95	89.8 × Root length (max M1)-219.4	
max M2		0.47 × Root length (max P4)-1.44	39.4 × Root length (max M2)-33.9	
**B: Mandible**				
	Estimated root length		Estimated root surface area	PPSA
	Supplement 2	Supplement 3	Supplement 4	Supplement 5
mandi I1		0.64 × Root length (max P4) + 2.55	13 × Root length (mandi I1) + 1.6	Estimated surface area × {1- (Estimated root length—Average PD_1-6_)^2^ / Estimated root length ^2^}
mandi I2		0.86 × Root length (max P4) + 0.26	14.8 × Root length (mandi I2) + 2.0	
mandi I3		0.59 × Root length (max P4) + 4.52	23.3 × Root length (mandi I3)-77.7	
mandi Canine		1.13 × Root length (max P4) + 5.92	40.6 × Root length (mandi Canine)-107.3	
mandi P1		0.29 × Root length (max P4) + 3.29	30.5 × Root length (mandi P1)-24.6	
mandi P2		0.41 × Root length (max P4) + 2.83	43.4 × Root length (mandi P2)-87.9	
mandi P3		0.45 × Root length (max P4) + 3.15	46.1 × Root length (mandi P3)-117.6	
mandi P4		0.52 × Root length (max P4) + 3.99	36.5 × Root length (mandi P4)-45.7	
mandi M1		0.78 × Root length (max P4) + 3.20	71.4 × Root length (mandi M1)-113.3	
mandi M2		0.25 × Root length (max P4) + 3.65	45.4 × Root length (mandi M2)-118.0	
mandi M3		0.82 × Root length (max P4)-5.36	17.7 × Root length (mandi M3) + 10.8	

*BW* body weight (kg) of dogs, *max* maxillary, *mandi* mandibular, *I* incisor, *P* premolar, *M* molar

**Fig. 2:**
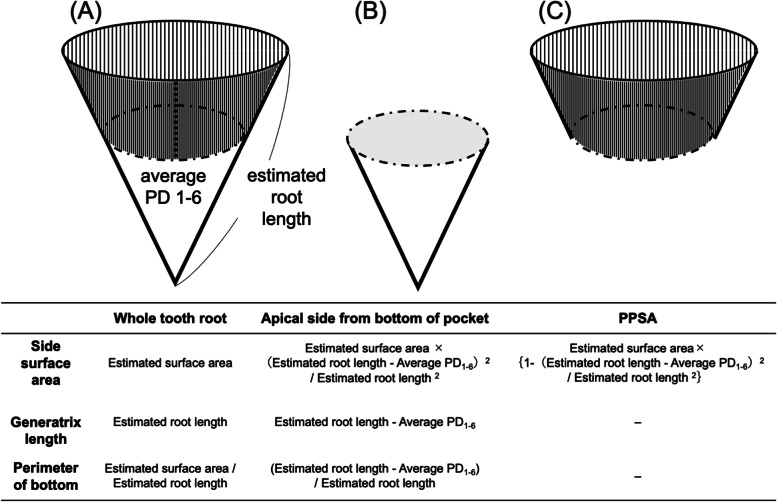
Scheme for calculating periodontal pocket surface area (PPSA) **A** whole tooth root; **B** apical side from the bottom of the pocket; **C** PPSA: the three parts of the cone (**A** to **C**) are used as the side surface area, generatrix length, and perimeter of the bottom, respectively. Meanings and formulas are shown in the table

The estimated root surface area is approximately equal to the side area of the cone, with the root length as the generatrix. The surface area of the periodontal pocket to be determined is an approximate value of the side area of the conical column, with the average value of the depth of the periodontal pocket measured using the six-point method as a generating line. Therefore, from the similarity ratio of the cone, the formula for the side area of the cone is the generatrix length × perimeter of the bottom. The apical side from the bottom of the pocket is expressed as the estimated surface area × (estimated root length – average PD_1-6_)^2^ / estimated root length^2^. The PPSA to be determined was the area obtained by subtracting it from the total root surface area. Thus, PPSA was expressed as [estimated surface area × {1 – (estimated root length – average PD_1-6_)^2^ / estimated root length^2^}] (Fig. 2). Finally, the PPSA estimation formula was established using body weight and periodontal pocket depth on an Excel-based spreadsheet (Supplement 5).

**Fig. 3:**
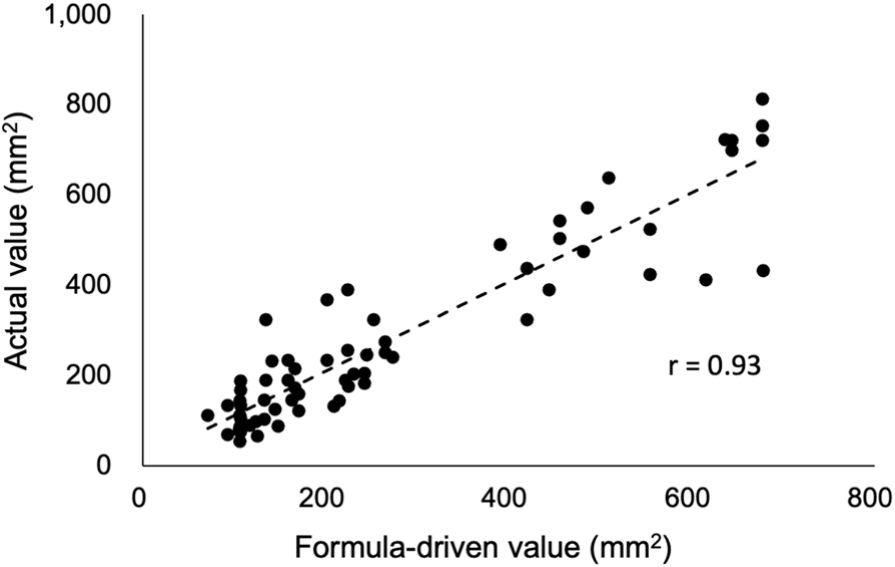
Scatter plot of the periodontal pocket surface area (PPSA) estimated by the formula and those of actual surface area obtained by tracing correlation coefficient using additional teeth (*n* = 66) is shown (*r* = 0.93,* p* < 0.01)

These data were applied to the root surface area of the extracted teeth (66 teeth) to evaluate the accuracy (Fig. 3). A high correlation coefficient was found between the estimated root surface area and the actual root surface area for 66 teeth (*r* = 0.93, *p* < 0.01). For other teeth grouped by number of roots, a strong correlation (*r* ≥ 0.8) was observed for PPSA (scatter plots in Supplement 6).

### Clinical application

The subjects aged 7–180 months (median: 132 months) and weighed 1.6–13.0 kg (median: 4.3 kg). The American Veterinary Dental College stages of periodontal disease (AVDC-SPD) were 1–4 (median: 4.0), and the surface area of the periodontal pocket was 30–9,333 mm^2^ (median: 2361 mm^2^).

Strong correlations between PPSA and serum globulin (*r* = 0.71) were found in the clinically applied data. In addition, moderate correlations of PPSA with C-reactive protein (CRP) (*r* = 0.54), packed cell volume (*r* = -0.43), and Alb (*r* = -0.56) were also observed. However, only a moderate correlation was found for AVDC-SPD with Glob (*r* = 0.43) (Table 6).

**Table 6:** Correlation coefficient of American Veterinary Dental College (AVCD) stages of periodontal disease and periodontal pocket surface area (PPSA) with each blood test

	AVDC-SPD (0–4)	*p*-value	PPSA (mm^2^)	*p*-value
PCV	-0.26	< 0.05	-0.43^b^	< 0.01
WBC	0.15	0.24	0.34	0.49
Plat	0.18	0.17	0.15	0.25
Glob	0.43	< 0.01	0.71^a^	< 0.01
Alb	-0.25	0.06	-0.56^b^	< 0.01
BUN	0.12	0.34	0.11	0.27
Cre	-0.06	0.66	0.16	0.46
ALT	0.11	0.39	-0.01	< 0.05
GGT	0.08	0.53	0.09	0.21
ALP	0.02	0.88	0.22	0.79
CRP	0.25	0.05	0.54^b^	< 0.01

Summary table of Supplement 7

*PCV* packed cell volume, *WBC* white blood cell, *Plat* platelet, *Glob* serum globulin, *Alb* serum albumin, *BUN* blood urea nitrogen, *Cre* creatine, *ALT* alanine transaminase, *GGT* γ-glutamyl transpeptidase, *ALP* alkaline phosphatase, *CRP* C-reactive protein

^a^strong correlation, ^b^moderate correlation

## Discussion

In this study, we established a method to estimate PPSA using the probing depth of periodontal pockets and body weight of dogs. Currently, PISA and PESA are clinically applied to estimate inflammation sites and pocket surface area in human dentistry; PPSA can be used for dogs similar to PESA in human dentistry. The accuracy of the obtained root surface area was verified by comparison with the actual root surface area, and the correlation coefficient between the measured and estimated values was more than 0.8 (*p* < 0.01) (Fig. 3). Furthermore, to investigate the relationship between the severity of periodontitis and systemic conditions, we examined the correlation between PPSA and blood test results before general anesthesia. We observed that some blood test results correlated with the severity of periodontitis, as indicated by PPSA (Table 6, Supplement 7). Thus, this study is the first to estimate the severity of periodontitis using PPSA and to show its relationship with blood test results.

In humans, PESA and PISA are calculated based on the root length and root surface area [13]. However, dogs are more diverse in body size [17] and tooth morphology than humans; thus, root length and root surface area must be calculated immediately to reflect the variation in body size. As described in the Materials and Methods section, we planned a strategy to estimate PPSA based on the probing depth to account for body weight. Max P4 was found to be an important tool for estimating root length, root surface area, and PPSA. Finally, the formula for calculating PPSA was established in three steps (Table 5) and summarized in an Excel-based spreadsheet (Supplement 5). Although the error due to the three-step estimation may be a concern, the nature of the regression equation is such the number of steps can be increased without being concerned about increasing the error. It is sufficient if the correlation coefficient and coefficient of determination can be maintained at a high level, even if the number of steps increases. This was confirmed by comparing the estimated root surface area obtained through the three steps with the root surface area obtained on actual measurement; it showed a high correlation with the actual measurement (Fig. 3). Therefore, we considered that the error would not be a problem in the final formula (Table 5, Supplement 5) after applying the three steps of this study. However, since we could not extract sufficient teeth owing to the small number of large-sized dogs in Japan, we need to devise methods to apply PPSA for large dogs and expand the range of indications.

Recently, measurement using digital imaging has become popular, and it is preferable to use three-dimensional (3D) scanning or cone beam computed tomography (CBCT) instead of the membrane method to measure the surface area of the root surface. However, the membrane method was chosen in this study since relatively expensive devices such as CBCT are not yet sufficiently popular in clinics. In addition, Wang et al. [18] reported that the distribution of values obtained using the membrane, 3D scan, and CBCT reconstruction methods was analyzed using the Kolmogorov–Smirnov test, and the results showed no significant difference, indicating that the membrane method is more reliable than other methods in measuring the root surface area of single- and multi-rooted teeth. They measured the membrane surface area by scanning and transforming digital data, but as suggested by Oda et al. [19], the manual membrane technique used in this study was useful.

**Fig. 4:**
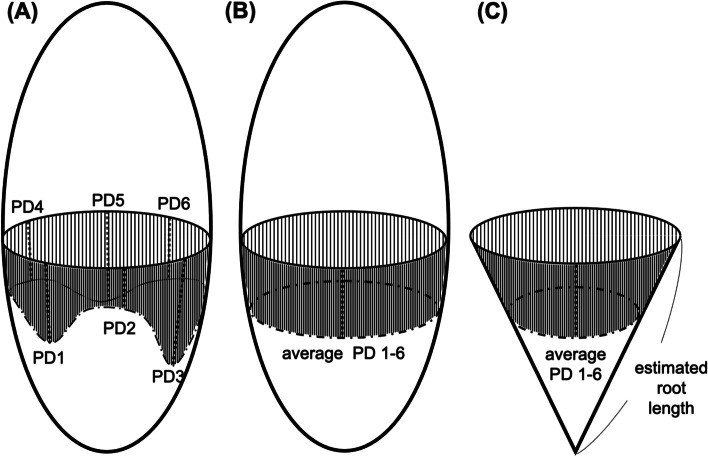
Periodontal pocket surface area (PPSA) estimation process **A** actual condition of the periodontal pocket, **B** equalization of the periodontal pocket depth by average calculation, and **C** estimation of the actual condition of the tooth surface as a virtual cone

Periodontal disease in dogs is similar to that in humans, with reactive hyperplasia associated with gingivitis [20] and gingival recession [21]. In this study, we considered the tooth as an oval (ellipse) and the root as an inverted cone (Fig. 4) and were thus able to calculate root length, root surface area, and their combinations as linear functions (Fig. 2; Supplements 3, 4, and 7). In fact, even PISA and PESA in humans are calculated based on an inverted cone morphology. Therefore, when the position of the gingival margin changes, errors will be less when periodontal pocket depth is measured than when attachment loss or gingival recession is measured [13]. Since periodontal pocket depth was measured in this study, the error in the change in gingival attachment was small.

When the relationship between periodontitis and general condition as considered in dentistry for humans was evaluated, we found strong correlations of PPSA with serum globulin (*r* = 0.71). In addition, moderate correlations of PPSA with CRP (*r* = 0.54), packed cell volume (*r* = -0.46), and serum albumin (*r* = -0.51) were also observed (Table 6). In this study, correlations with several blood investigations were confirmed for the existing periodontal disease classification and established PPSA. In a previous study based on total mouth periodontal score (TMPS) evaluation [22], a decrease in Plat and Cre was detected accordingly. The difference between these studies was in terms of the range of body weights considered. In contrast to the weight range in the previous report (2–39 kg [median: 16 kg]), the range in our study was 1.6–13.0 kg (median: 4.3 kg). The methods used to estimate the surface area of periodontal pockets were also different. According to multiple reports [23–25], severe periodontitis may be more prevalent in small dogs than in large dogs, although a high prevalence of periodontitis in any size of dogs has been reported previously. Therefore, the present study may have included dogs with a more severe periodontal condition than that previously reported, which correlated with results of many blood tests, suggesting that the expansion of periodontal disease causes systemic inflammation as indicated with CRP and Glob data. Histopathological abnormalities in the kidney and liver have been reported in dogs that died of periodontal and other diseases [26]; however, the present results did not show any abnormal data for these organs. Since our study was an observational study at a single institution, a prospective multicenter study is necessary to clarify the pathogenesis of periodontal disease, its onset, and exacerbation.

In this study, the bleeding on probing (BOP) of each tooth was not recorded in the medical record, thus the pseudo-area of periodontal pocket (PPSA) was evaluated instead of the degree of periodontal inflammation (PISA). For future clinical application, it is necessary to record the BOP of each tooth in order to measure both PPSA and PISA in dogs. In the PPSA estimation method established in this study, PISA in dogs can be easily calculated by adding positive BOP points as one of the representative gingival bleeding indices, as in the case of calculating PISA in humans. In conclusion, we succeeded in constructing a formula for calculating PPSA to understand the pathology of periodontal disease in small- and medium-sized dogs and to determine its severity. In the future, the addition of data of large-sized dogs and the use of dogs’ PISA considering BOP-positive areas may expand our understanding of periodontitis-related systemic diseases.

## Methods

### General strategy to establish the formula for estimating ppsa

**Fig. 5:**
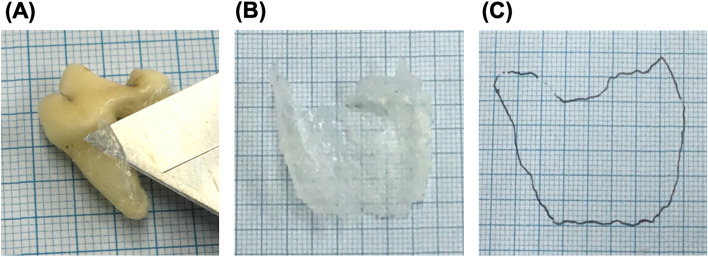
Measurement method of the root surface area **A** a thin film from the tooth root cut with a knife, **B** a thin film placed on a 1-mm grid paper, and **C** yielded root surface area after tracing

We hypothesized that PPSA resembles the surface of a cone, as shown in Fig. 4. Periodontal pocket depth was obtained as the average pocket probing depth at the six probing points (Fig. 4A, 4B). The morphology of the tooth root was deemed as a cone, and the generatrix from base to apex was considered as the length of the tooth root (Fig. 4C). In addition, the periodontal pocket was deemed as a circular truncated cone with a generatrix from the top to bottom considered as pocket probing depth (Fig. 4C). The side surface areas of the cone and circular truncated cone were considered as the root surface and PPSA, respectively (Fig. 4C). To utilize this approximate form to establish the formulas (regression equation) to estimate the length and surface of the tooth root, actual values were obtained by measuring them using extracted teeth (Supplement 1 and Fig. 5).

The root length of each extracted tooth to the body weight of the dog was compared, and the correlation and formula were thus obtained. Initially, the formula to estimate actual root length from actual body weight (Supplement 2) was obtained, with max P4 showing the highest correlation (≥ 0.70) and other teeth showing a correlation < 0.70. Furthermore, the root length of other teeth was estimated using the corresponding formula for the root length of max P4 with each dog’s weight (Supplement 3).

The formula to estimate the root surface area from the root length of each tooth type was obtained by comparing their actual values (Supplement 4). Using this formula, the root surface could be estimated using the formula for the calculated root length corresponding to each dog’s weight. Finally, PPSA was determined by subtracting the apical surface area from the root surface area (Fig. 2).

After completing the above process and inputting the weight of the case, the root length of max P4 can be estimated using the estimation formula. Based on this value, the root lengths of other tooth types can be estimated accordingly. Since the root surface area can be estimated from all root lengths and PPSA is expressed as [estimated surface area x {1-(estimated root length-average PD1-6)^2^/estimated root length^2^}], the weight of the case can be input to calculate PPSA.

### Characteristics of tooth morphology and donor dogs

Extracted teeth (625 teeth from 73 dogs of 16 breeds) were collected from eight veterinary clinics in Japan, and the root length and root surface area of extracted teeth were measured accordingly. A silk thread (#0; Nesco suture® silk suture, Allfresa Pharma Co., Ltd., Osaka, Japan) was placed from the dento-enamel junction at the cervix to the root apex along the vertical axis of the extracted teeth, and its length was measured. The average length of multiple roots was used for multi-rooted teeth.

The measurement method of the root surface area was modified from that used for extracted human teeth [19]. The extracted teeth were immersed in a surfactant (Rookie Fresh V, Daiichi Soap Co., Ltd., Gunma, Japan) for 30 min, and the remaining surfactant on the root surface was dried. Thereafter, ethyl acetate (vinyl acetate resin, an emulsion type adhesive for bond woodworking; Konishi Co., Ltd., Osaka, Japan) was applied, and the teeth were dried for 12 h. Cyanoacrylate (Aron Alpha, Konishi Co., Ltd.) was used for lamination. The cyanoacrylate-laminated thin film was incised using a knife and then peeled for collection. Thereafter, the edge of this thin film was traced on a 1-mm square graph sheet, and its surface area was measured accordingly (Fig. 5).

In addition, the body weight of the donor dog of the extracted tooth was obtained from medical records. These data are summarized in Supplement 1.

### Estimation formula for root length correlated with body weight

The correlation was evaluated to create a regression equation for estimating the root length from body weight. The scatter plot between actual body weight and actual root length was drawn for each tooth type, and both coefficient and linear equations for estimating root length (y) were determined using body weight (x) as a variable (Supplement 2). Then, a formula (y = ax + b) was determined for the strongest correlation coefficient with body weight (*r* ≥ 0.7). Furthermore, scatter plots between the actual and estimated root lengths of each tooth with the strongest correlation coefficient with the body weight mentioned above were drawn. Then, the second formula (z = cy + d; constants ‘c’ and ‘d’ specific for each tooth type) was obtained to estimate the root length of other tooth (z) by considering the body weight of each dog in place of ‘x’ (Supplement 3).

### Estimation formula for root surface area correlated with root length

The correlation was evaluated to create a regression equation for estimating the root surface area from the estimated root length. The scatter plot between actual root length and actual root surface area was drawn for each tooth type, and a formula (α  = ez + f; constants ‘e’ and ‘f’ specific for each tooth type) for estimating root surface area (α) was determined using the root length (z) estimated using the formula mentioned above (Supplement 4).

### Estimation formula for periodontal pocket surface area

PPSA was calculated as the side area of a conical column with the mean value of the periodontal pocket depth measured using the six-point method as the generatrix. The relational formula was obtained from the similarity ratio for the side area of the conical column with the average value of the periodontal pocket depth as the generating line with respect to the root surface area and with the estimated root length as the generating line (Fig. 2).

### Validation of the formula using additional extracted teeth

Additional extracted teeth (66 teeth) were obtained randomly from six dogs (five breeds) at one of the veterinary clinics (raw data in Supplement 6). The actual values of root surface and body weight were obtained and used to validate the formula.

### Clinical application

The PPSA formula was applied to 61 periodontitis-related dogs under anesthesia at one of the veterinary clinics. As a regular procedure, preoperative blood tests and assessment of periodontal disease based on the classification of AVDC-SPD [27] were performed (Supplement 7). PPSA estimation using the Excel-based spreadsheet with the formula (Supplement 5) was applied to evaluate the severity of periodontitis in addition to the classification of AVDC-SPD.

All dogs were uniformly treated with periodontal surgery using analgesics. Some were maintained in this state using analgesics (oral robenacoxib 0.8–1.2 mg/kg bid body weight once daily for 2 days), and oral antibiotics (oral amoxicillin 11–22 mg/kg bid body weight twice daily for 6 days) were administered. Blood investigations were performed a week before and a day before the surgery. In addition, to confirm that cases did not have any major inflammation outside of the oral cavity, after 2–4 weeks, blood tests were repeated to confirm whether the abnormal values normalized. Blood samples dispensed into ethylenediamine tetra-acetic acid (EDTA) tubes were used for determining packed cell volume (PCV), white blood cell (WBC), and platelets (Plat) counts with a multi-item automatic blood cell calculator (pocH®-100iV, Sysmex Corporation, Hyogo, Japan). Blood samples dispensed into serum separation tubes were used for biochemical examinations such as Glob, albumin (Alb), blood urea nitrogen (BUN), creatinine (Cre), alanine transaminase (ALT), alkaline phosphatase (ALP), and γ-glutamyl transpeptidase (GGT) using in-house biochemical test equipment (IDEXX Catalyst One, IDEXX Laboratories, Tokyo, Japan; Supplement 7). CRP levels were also measured (Laser CRP-2; Arrows Co., Ltd., Osaka, Japan). Periodontitis was classified according to AVDC-SPD using a diagnostic X-ray apparatus (RADspeed Pro, Shimadzu Corporation Co., Ltd., Kyoto, Japan) and D sensitivity instant film (ISO speed D X-ray dental film, Hanshin Technical Laboratory, Ltd, Hyogo, Japan). Full-mouth intraoral X-ray radiography was performed under anesthesia to evaluate the degree of alveolar bone resorption. The tooth with the most severe resorption of the alveolar bone in each case was classified according to AVDC-SPD. Periodontal pocket depth was measured with a periodontal probe (Perio probe #5, YDM Co., Ltd., Tokyo, Japan) by the first author (KT) only and used for calculation of PPSA and evaluation of correlations with each blood test data.

### Statistical analysis

The correlation coefficient and regression equation were calculated using body weight and the root length of each extracted tooth type (Supplement 2), using the root length of the tooth type most correlated with body weight and the root length of another tooth type (Supplement 3), and using the root length and root surface area of each tooth type (Supplement 4). Correlations between either AVDC-SPD or PPSA and various blood test data were then calculated (Supplement 7).

For all correlation tests, the correlation coefficient was calculated using the Pearson function, and the p-value was calculated using the TDIST function. The median body weight of donor dogs whose extracted teeth were used and body weight of dogs for clinical application were calculated using the median function.

All analyses were performed using Microsoft Excel for Mac (Version 16.43).

## Supplementary Information

**Additional file 1.** Distribution of extracted tooth number, root length, and body weight of each breed.

**Additional file 2.** For each tooth type, comparison of the actual root length and actual body weight.

**Additional file 3.** For each tooth type, comparison of the actual root length and estimated value using the formula for the maxillary fourth premolar root length.

**Additional file 4.** For each tooth type, comparison of the actual root surface area and estimated root length using the formula for the maxillary fourth premolar root length.

**Additional file 5.** The periodontal pocket surface area estimation formula was established using body weight and periodontal pocket depth on an Excel-based spreadsheet.

**Additional file 6.** Accuracy of the root surface area validated using an Excel-based spreadsheet to the actual root surface area.

**Additional file 7.** Clinical application data. Some blood test results correlated with the severity of periodontitis shown using periodontal pocket surface area.

## Data Availability

The dataset generated and/or analyzed during the current study is available from the corresponding author on reasonable request.

## Abbreviations

3D: Three-dimensional
Alb: Albumin
ALP: Alkaline phosphatase
ALT: Alanine transaminase
AVDC-SPD: American Veterinary Dental College stages of periodontal disease
BOP: Bleeding on probing
BUN: Blood urea nitrogen
CBCT: Cone-bean computer tomography
Cre: Creatine
CRP: C-reactive protein
GGT: G-glutamyl transpeptidase
Glob: Serum globulin
I: Incisor
M: Molar
Mandi: Mandibular
Maxi: Maxillary
n: Number of extracted teeth
ND: Not determined as treated as standard
PM: Premolar
PCV: Packed cell volume
Plat: Platelets
PISA: Periodontal inflamed surface area
PPSA: Periodontal pocket surface area
TMPS: Total mouth periodontal score
WBC: White blood cell
